# Impact of self-reported SARS-CoV-2 antibody positivity on cardiac structure and function: findings from UK Biobank CMR cohort

**DOI:** 10.3389/fcvm.2025.1462263

**Published:** 2025-02-27

**Authors:** Chang Liu, Yao Ma, Shiyuan Qiao, Kexin Li, Mengyao Qi, Chunyu Gu, Lanxin Zhang, Jia Wei, Dengfeng Gao

**Affiliations:** ^1^ Department of Cardiology, The Second Affiliated Hospital of Xi’an Jiaotong University, Xi’an, China; ^2^ Department of Radiology, Affiliated Hospital of Shaanxi University of Chinese Medicine, Xianyang, China

**Keywords:** UK Biobank, COVID-19, cardiovascular magnetic resonance cohort, native T1, left ventricular hypertrophy

## Abstract

**Background:**

SARS-CoV-2 antibody positivity, whether due to natural infection or vaccination, is known to be associated with specific cardiac and vascular damage, yet its impact on cardiac structure and function in prospective cohorts remains incompletely understood.

**Objective:**

We aimed to assess cardiac changes in the UK Biobank cohort among individuals with self-reported seropositive results for SARS-CoV-2 antibodies.

**Methods:**

UK Biobank participants with self-reported serological results for SARS-CoV-2 antibodies, who underwent their first cardiac magnetic resonance (CMR) scan after 2019 were included. Cardiac changes potentially associated with SARS-CoV-2 antibody positivity were assessed, with measurements of left ventricular (LV) parameters, including volume, dimensions, wall thickness, myocardial mass, cardiac output (CO), and cardiac index (CI), manually extracted from the CMR images. Propensity score matching (PSM) was used to pair seropositive and seronegative individuals. Native T1 was used to assess the within-subject changes in seropositive individuals. Logistic regression was performed to assess the association between SARS-CoV-2 antibody status and the incidence of LV hypertrophy.

**Results:**

A total of 720 participants were included, with 453 individuals self-reporting as SARS-CoV-2 antibody positive. After PSM, 261 participants remained in each group. Over an average follow-up period of 110 days, significant decreases in CO and CI were observed in the paired participants. Additionally, native T1 values appeared to be elevated in seropositive participants (852.77 ± 53.55 ms vs. 860.01 ± 47.81 ms, *P* = 0.012). Logistic regression analysis in the overall cohort indicated an association between SARS-CoV-2 antibody positivity and an increased risk of LV hypertrophy, with an adjusted odds ratio of 3.257 [95% CI (1.036–10.239), *P* = 0.043].

**Conclusions:**

Our findings suggest subtle cardiac changes associated with SARS-CoV-2 antibody positivity within approximately hundred days. SARS-CoV-2 antibody positivity appeared to be associated with an increased risk of LV hypertrophy. However, these results are exploratory, and further longitudinal studies with extended follow-up are needed to better understand the long-term cardiac impact of SARS-CoV-2 antibody positivity.

## 1 Introduction

As the Coronavirus disease 2019 (COVID-19) pandemic persists worldwide, Severe Acute Respiratory Syndrome Coronavirus 2 (SARS-CoV-2) not only targets the respiratory system through the angiotensin-converting enzyme 2 receptor (1), but also inflicts damage on cardiovascular system, leading to myocardial injury (2).

Concurrently, the numerous sequelae of SARS-CoV-2 infection have led to a significant increase in cardiovascular adverse events, highlighting the crucial need for ongoing monitoring and management of cardiovascular health in patients recovering from COVID-19 (3–7). Moreover, there have been reports of cardiac side effects following COVID-19 vaccination, including myocarditis and pericarditis, particularly in younger individuals. These adverse events further highlight the importance of monitoring cardiac health not only in those infected with SARS-CoV-2 but also in vaccinated individuals (8, 9).

Cardiovascular magnetic resonance (CMR) examination serves as an essential and valuable tool for assessing myocardial structure and cardiac function. Advanced CMR techniques such as native T1 mapping, extracellular volume (ECV) fraction, and late gadolinium enhancement (LGE) enable the detection of subtle changes in myocardial tissue. The American College of Cardiology, the European Society of Cardiology, and the Society for Cardiovascular Magnetic Resonance have collectively acknowledged the value of CMR in evaluating the structural and functional repercussions of SARS-CoV-2 infection (10, 11).

Long COVID, characterized by persistent symptoms and long-term complications following acute SARS-CoV-2 infection, has also been associated with significant cardiovascular issues (12). The UK Biobank conducted a monthly follow-up survey for COVID-19 patients within its cohort. From May to November 2020, among the 10,878 COVID-19 patients surveyed, a subset exhibited symptoms suggestive of cardiovascular involvement, including shortness of breath (4.30%), wheezing (2.21%), chest pain (1.84%), nausea (1.93%), and increased fatigue (10.08%). Of these patients, 3.37% required medical intervention for COVID-19-related symptoms, and 0.21% required hospitalization.

A cross-sectional study of CMR parameters in SARS-CoV-2 seropositive patients from the UK Biobank cohort revealed that, beyond traditional cardiovascular risk factors, pre-existing adverse CMR phenotypes may be associated with susceptibility to COVID-19 (13). With the gradual release of subsequent CMR examinations conducted after 2019 for repeat imaging visits by the UK Biobank, we are now able to investigate whether there are any subtle changes in CMR parameters among participants with self-reported positive SARS-CoV-2 antibody test results.

## 2 Methods

### 2.1 Study design and participants

The UK Biobank established a population-based cohort study, recruiting 502,357 participants aged 39 to 70 years at baseline from 2006 to 2010. Among these, 69,902 participants underwent at least one long axis heart CMR scan(data field 20208), while a total of 5,154 participants completed a first repeat CMR scan, and 811 of these participants had their initial CMR scan after January 1, 2019. Participants for this study were selected based on having both initial and follow-up CMR scans and at least one SARS-CoV-2 antibody test result.

**Figure 1:**
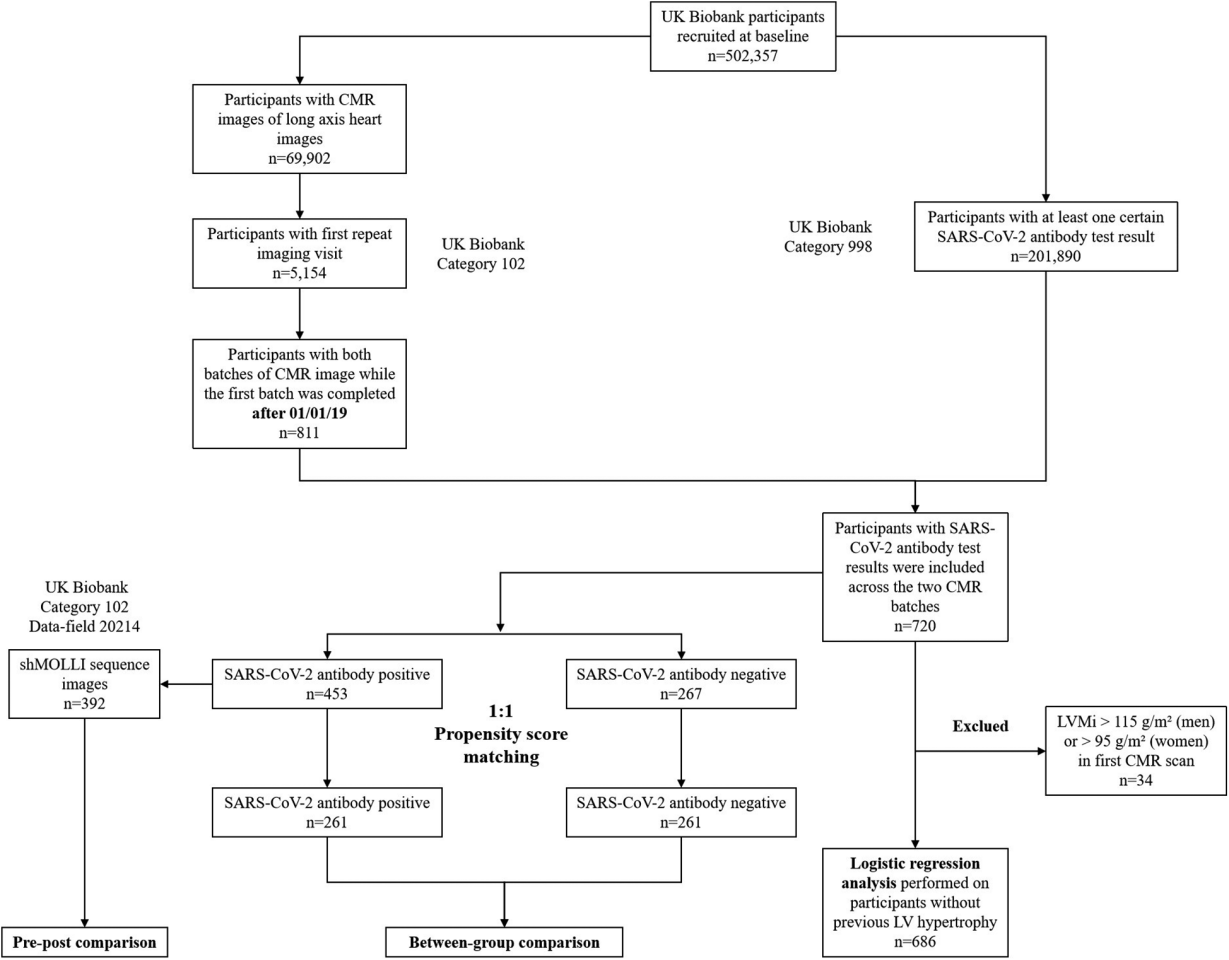
Flow chart of participant selection and experimental procedures.

To ensure representative and unbiased results, we selected participants who had completed their first CMR scan after January 1, 2019, and whose antibody test results were available between the initial and follow-up scans. The participant selection process is illustrated in Figure 1. This selection process ensured that the study cohort included individuals who had both pre- and post-SARS-CoV-2 antibody test result data.

Additionally, 201,890 participants had at least one self-reported SARS-CoV-2 antibody test result (data field 27,981), of which 720 participants' first antibody test result was obtained between the initial and follow-up CMR scans.

Among the 453 seropositive participants, 392 had experimental shortened Modified Look-Locker Inversion Recovery (shMOLLI) sequence images (data field 20,214). After performing 1:1 propensity score matching (PSM), 261 participants were retained in both the SARS-CoV-2 antibody positive and negative groups.

A list of International Classification of Disease (ICD) codes used to define baseline diseases is provided in Supplementary Table S1 (data field 41,270).

### 2.2 SARS-CoV-2 antibody test

Participants were initially categorized as positive or negative based on self-reported results using the Fortress Rapid Test kit or AbC-19TM Rapid Test kit between February 2021 and July 2021. These antibody results were collected over two rounds during this six-month period. The data were subsequently compiled and made available by the UK Biobank Participant Resource Centre. For the analysis, the first available result for each participant was used to determine whether they tested positive for SARS-CoV-2 antibodies. The antibody results obtained in this manner were collected at least one year after the initial CMR scan of the imaging follow-up cohort included in the study.

### 2.3 CMR scanning protocol and cardiac parameters

The UK Biobank plans to recall approximately 100,000 participants for a comprehensive CMR examination as part of their multi-organ, multi-modality imaging visit (14). The CMR protocol was previously described in detail, and in summary, all steady-state free precession cine imaging of CMR long axis images were conducted using a 1.5 Tesla scanner, with no MR-contrast enhancement for safety reasons (15). CMR image analysis was performed by two radiologists specializing in MRI and one cardiovascular specialist using cvi42 image post-processing software (Version 5.11, Circle Cardiovascular Imaging Inc., Calgary, Canada). Manual contouring was employed to extract left ventricular volumes, myocardial mass, and dimensions during both end-diastolic and end-systolic phases from the long axis images. LV myocardial native T1 was manually extracted from the experimental shMOLLI sequence on a mid-ventricular short-axis image.

Our focus was on various LV parameters, including left ventricular end-diastolic volume (LVEDV), end-systolic volume (LVESV), stroke volume (LVSV), ejection fraction (LVEF), cardiac output, cardiac index, left ventricular mass (LVM), end-diastolic dimension (Dd), end-systolic dimension (Ds), posterior wall thickness (PWT), relative wall thickness (RWT), left ventricular mass index (LVMi), left ventricular global function index (LVGFi), left ventricular mass volume ratio (LVMVR) on long axis heart images, and myocardial native T1 on short axis shMOLLI sequence images. LV hypertrophy was defined as LVMi greater than 115 g/m^2^ (for men) and 95 g/m^2^ (for women).

For the imaging analysis, CMR parameters were extracted from the first and repeat CMR tests for all 5,154 participants in a single batch. Analysts were blinded to participants' SARS-CoV-2 antibody status and personal information to prevent bias. To ensure consistency, an intra-class correlation coefficient (ICC) analysis was conducted between the three observers on the first 2,000 cases, demonstrating excellent internal consistency with all ICC values exceeding 0.75. The results for the remaining cases were obtained through consensus among the three observers, with the final analysis completed by the cardiovascular specialist.

### 2.4 Statistics analysis

Statistical analysis was conducted using Python 3.9.13. Continuous variables were presented as mean (standard deviation) or median (interquartile range), while categorical variables were expressed as percentages. The normality distribution was assessed using the Kolmogorov–Smirnov test. Baseline characteristics and CMR parameters were compared using the two-sample *t*-test for normally distributed variables or the Wilcoxon rank-sum test for non-normally distributed variables, and the chi-square test for categorical variables.

We employed PSM to randomly select a matched control participant for each seropositive participant. The PSM caliper value was set at 0.05. The PSM considered factors including age, sex, ethnity, TDI (Townsend Deprivation Index), height, weight, BMI (Body Mass Index), BSA (Body Surface Area), SBP (Systolic Blood Pressure), DBP (Diastolic Blood Pressure), current smoking, current drinking, self-reported history of hypertension, hyperlipidemia, diabetes, asthma, and other cardiovascular diseases. Paired *t*-tests were performed after PSM.

Logistic regression analysis was performed to evaluate the association between SARS-CoV-2 antibody status and the incidence of LV hypertrophy in the overall study population, with adjustment for relevant covariates. Participants with LV hypertrophy detected in their initial CMR scan were excluded from further analysis. Model 1 comprised a univariate analysis, Model 2 incorporated adjustments for age, sex, ethnity, TDI, height, weight, BMI, BSA, SBP, DBP. Model 3 was further adjusted for a history of hypertension, cardiovascular disease, and diabetes. A *P*-value < 0.05 was considered statistically significant.

## 3 Results

### 3.1 Characteristics of study participants

With the gradual release of subsequent CMR examinations conducted after 2019 for repeat imaging visit by the UK Biobank, we are now able to investigate the variations in CMR parameters among participants with different SARS-CoV-2 antibody status. A total of 720 participants were included, of which 453 self-reported as SARS-CoV-2 seropositive. All participants underwent their initial CMR imaging after 2019, followed by subsequent CMR examinations after the detection of SARS-CoV-2 antibody results. Table 1 presents the clinical characteristics of the study participants. Significant differences in age were observed between the two groups. After 1:1 PSM, 261 participants remained in each group. The clinical characteristics post-matching, stratified by SARS-CoV-2 antibody results, are presented in Table 2. Following PSM, no significant differences were observed between the groups in terms of other baseline characteristics, except for vaccination status. The median interval between the date of SARS-CoV-2 antibody testing and the follow-up CMR examination was 109 days for the seropositive group and 112 days for the seronegative group.

**Table 1:** Baseline characteristics of study participants.

Baseline characteristics	SARS-CoV-2 antibody positive *n* = 453	SARS-CoV-2 antibody negative *n* = 267	*P* value
Age (year)	52 (11)	50 (12)	0.001^b^
Male (%)	49.4	45.9	0.361^c^
TDI	−1.97 (4.60)	−1.95 (4.27)	0.629^b^
Weight (kg)	77.69 (13.55)	77.76 (14.88)	0.944^a^
Height (cm)	170.81 (9.39)	171.55 (9.20)	0.307^a^
BMI (kg/m^2^)	1.88 (0.21)	1.89 (0.23)	0.744^a^
BSA (m^2^)	1.9 (0.2)	1.9 (0.2)	0.782^a^
SBP (mmHg)	139 (18)	139 (20)	0.491^b^
DBP (mmHg)	78 (11)	78 (11)	0.716^b^
Days interval (day)	128 (109)	120 (108)	0.553^b^
Vaccinated (%)	81.8	61.7	<0.001^c^
Ethnity			
White (%)	92.5	90.3	0.311^c^
Other ethnicities (%)	7.5	9.7	–
Life-style			
Current Smoking (%)	39.1	40.1	0.790^c^
Current drinking (%)	94.9	94.0	0.600^c^
Self-reported diseases			
Hypertension (%)	13.7	12.4	0.611^c^
High Cholesterol (%)	7.7	6.4	0.496^c^
Diabetes mellitus (%)	5.3	5.2	0.975^c^
Asthma (%)	6.0	6.0	0.986^c^
Cardiac diseases (%)	6.8	8.6	0.384^c^

Continuous variables were summarized using means and standard deviations or medians and interquartile ranges, categorical variables were summarized using percentages.

Ab, antibody; TDI, townsend deprivation score; BMI, body mass index; BSA, body surface area; SBP, systolic blood pressure; DBP, diastolic blood pressure.

^a^ Two-sample *T*-test.

^b^ Wilcoxon rank sum test.

^c^ chi square test.

**Table 2:** Baseline characteristics of study participants after 1:1 propensity score matched with SARS-CoV-2 antibody test result.

Baseline characteristics	SARS-CoV-2 antibody positive *n* = 261	SARS-CoV-2 antibody negative *n* = 261	*P-*value
Age (year)	51 (11.5)	50 (12)	0.628^b^
Male (%)	47.5	49.0	0.726^c^
TDI	−2.20 (4.68)	−1.95 (4.23)	0.972^b^
Weight (kg)	77.67 (13.73)	77.70 (14.85)	0.986^a^
Height (cm)	170.84 (9.41)	171.43 (9.22)	0.471^a^
BMI (kg/m^2^)	1.88 (0.21)	1.89 (0.23)	0.843^a^
BSA (m^2^)	1.9 (0.2)	1.9 (0.2)	0.782^a^
SBP (mmHg)	139 (18)	139 (19)	0.608^b^
DBP (mmHg)	78 (11)	78 (11)	0.614^b^
Days interval (day)	109 (115)	120 (107)	0.522^b^
Vaccinated (%)	77.3	62.4	<0.001^c^
Ethnity			
White (%)	92.0	92.3	0.871^c^
Other ethnicities (%)	8.0	7.7	–
Life-style			
Current Smoking (%)	37.9	39.5	0.719^c^
Current drinking (%)	93.9	94.3	0.853^c^
Self-reported diseases			
Hypertension (%)	11.9	11.9	1.000^c^
High Cholesterol (%)	5.7	5.7	1.000^c^
Diabetes Mellitus (%)	4.2	5.4	0.539^c^
Asthma (%)	5.7	6.1	0.853^c^
Cardiac diseases (%)	8.0	7.3	0.742^c^

Continuous variables were summarized using means and standard deviations or medians and interquartile ranges, categorical variables were summarized using percentages.

Abbreviations as in Table 1.

^a^ Paired *T*-test.

^b^ Wilcoxon rank sum test.

^c^ Chi square test.

### 3.2 Cardiac parameters of initial and follow-up CMR cohort

After 1:1 PSM, seropositive participants exhibited lower LVEDV, LVESV, LVEDVi, LVESVi, Dd, and Ds during the initial CMR scan compared to the negative group. These differences persisted during the subsequent CMR test, with seropositive participants still showing lower LVEDV, LVESV, LVEDVi, and LVESVi (Table 3).

**Table 3:** Comparison of LV parameters in initial and subsequent follow-up CMR examinations between PSM-processed SARS-CoV-2 antibody positive and negative groups.

LV parameters	Group A (Initial, positive)	Group B (Subsequent, positive)	Group C (Initial, negative)	Group D (Subsequent, negative)	*P*-value			
					A/B (Intra-group)	C/D (Intra-group)	A/C (Inter-group)	B/D (Inter-group)
LVEDV (ml)	116.94 (26.67)	116.45 (29.03)	122.95 (38.69)	117.95 (39.67)	0.680	0.313*	0.007	0.041
LVESV (ml)	30.11 (11.21)	31.08 (13.45)	32.30 (16.83)	31.41 (18.87)	0.133	0.853*	0.004	0.036
LVEDVi (ml/m^2^)	61.99 (11.83)	61.84 (13.22)	65.47 (12.33)	64.73 (13.04)	0.802	0.508	0.001	0.011
LVESVi (ml/m^2^)	16.01 (5.66)	16.52 (6.78)	17.30 (8.29)	17.16 (8.63)	0.130	0.969*	0.002	0.027
LVSV (ml)	86.83 (20.16)	85.49 (20.36)	88.82 (27.68)	87.25 (26.06)	0.152	0.199*	0.068	0.145
LVEF (%)	74.46 (6.87)	73.98 (7.20)	73.51 (7.44)	73.09 (9.51)	0.270	0.519*	0.106	0.110
CO (l/min)	5.38 (1.30)	5.09 (1.24)	5.33 (1.74)	4.92 (1.60)	<0.001	0.002*	0.746	0.787
CI (l/min/m^2^)	2.85 (0.58)	2.60 (0.66)	2.83 (0.81)	2.65 (0.67)	<0.001	0.003*	0.667	0.558*
LVM (g)	150.82 (53.45)	145.29 (56.64)	153.35 (55.81)	152.03 (60.09)	0.977*	0.778*	0.322	0.380*
Dd (mm)	50.87 (5.24)	50.96 (5.43)	52.18 (5.21)	51.68 (5.35)	0.742	0.280	0.003	0.096
Ds (mm)	28.34 (4.38)	28.40 (4.56)	29.23 (4.66)	29.22 (4.63)	0.769	0.974	0.012	0.052*
PWT (mm)	10.23 (1.98)	10.41 (1.97)	10.36 (3.06)	10.11 (2.44)	0.105	0.461*	0.612	0.428
RWT	0.39 (0.10)	0.40 (0.13)	0.39 (0.13)	0.39 (0.11)	0.522*	0.969*	0.257*	0.136
LVMi (g/m^2^)	78.91 (19.08)	79.85 (19.46)	82.24 (20.61)	81.35 (21.54)	0.978*	0.811*	0.156*	0.163*
LVGFi	0.40 (0.06)	0.39 (0.06)	0.40 (0.06)	0.39 (0.08)	0.095	0.440*	0.780	0.704
LVMVR	1.30 (0.32)	1.29 (0.39)	1.26 (0.31)	1.23 (0.33)	0.976	0.850*	0.082	0.146*

Group A—Initial batch of SARS-CoV-2 antibody positive participants; Group B—Subsequent batch of SARS-CoV-2 antibody positive participants; Group C—Initial batch of SARS-CoV-2 antibody negative participants; Group D—Subsequent batch of SARS-CoV-2 antibody negative participants. Independent variables was calculated as means and standard deviation or median and interquartile range.

LVEDV, left ventricular end-diastolic volume; LVESV, left ventricular end-systolic volume; LVSV, left ventricular stroke volume; LVEF, left ventricular ejection fraction; CO, cardiac output; CI, cardiac index; Dd, end-diastolic dimension; Ds, end-systolic dimension; PWT, posterior wall thickness; RWT, relative wall thickness; LVM, left ventricular mass; LVGFi, left ventricular global function index; LVMVR, left ventricular mass-volume ratio.

* Indicates results from the Wilcoxon test.

**Figure 2:**
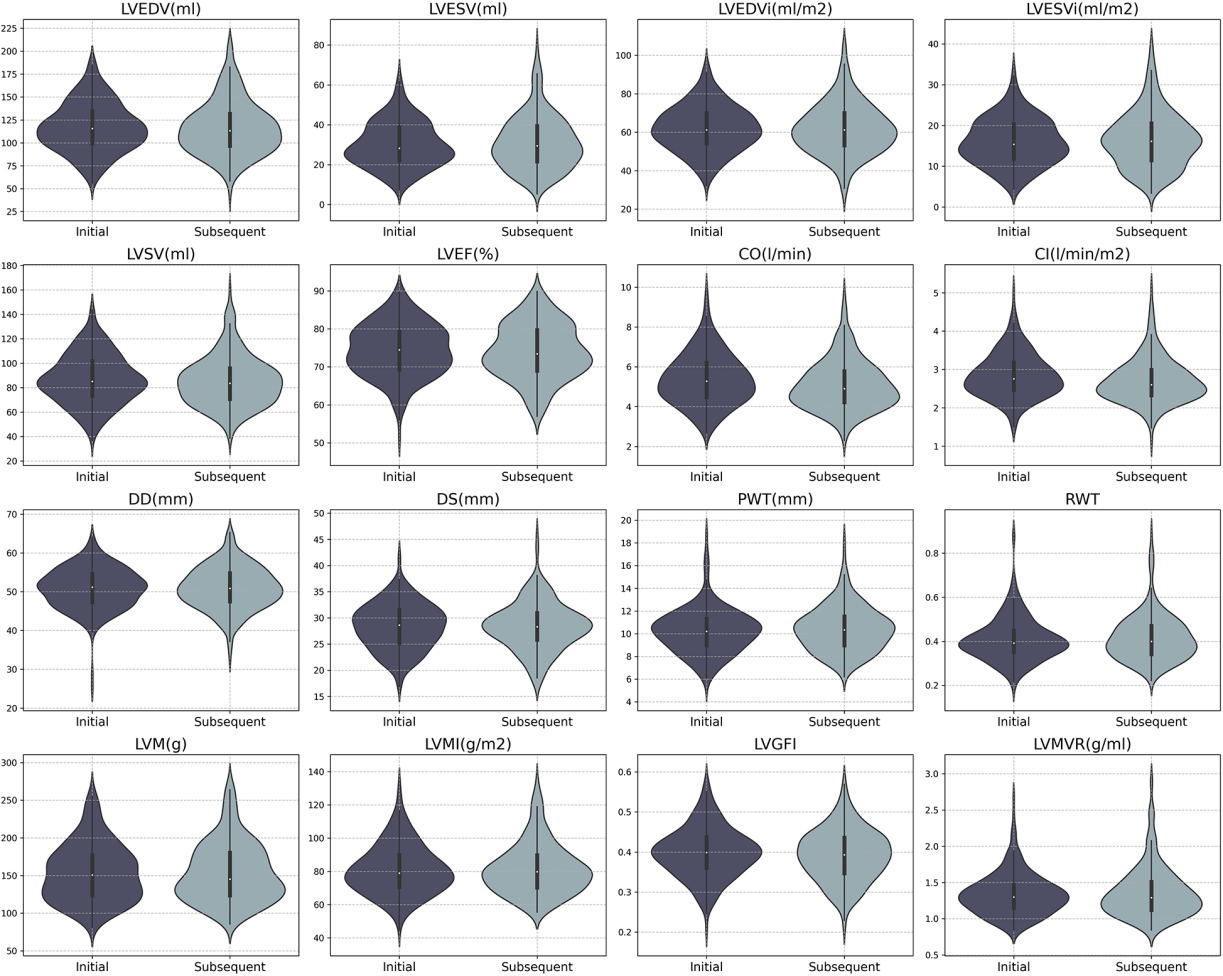
Distribution of CMR parameters in initial and subsequent batches for SARS-CoV-2 antibody positive participants. LVEDV, left ventricular end-diastolic volume; LVESV, left ventricular end-systolic volume; LVSV, left ventricular stroke volume; LVEF, left ventricular ejection fraction; CO, cardiac output; CI, cardiac index; Dd, end-diastolic dimension; Ds, end-systolic dimension; PWT, posterior wall thickness; RWT, relative wall thickness; LVM, left ventricular mass; LVGFi, left ventricular global function index; LVMVR, left ventricular mass-volume ratio.

In all seropositive participants, significant reductions were observed in LV parameters, including CO and CI following the onset of antibody positivity (mean ± SD: CO 5.38 ± 1.30 L/min vs. 5.09 ± 1.24 L/min, *P* < 0.001; CI 2.85 ± 0.58 L/min/m^2^ vs. 2.60 ± 0.66 L/min/m^2^, *P* < 0.001, see Table 3 and Figure 2). However, a similar declining trend was evident in the negative group (median (IQR): CO 5.33 (1.74) L/min vs. 4.92 (1.60) L/min, *P* = 0.002; CI 2.83 (0.81) L/min/m^2^ vs. 2.65 (0.67) L/min/m^2^, *P* = 0.003).

**Figure 3:**
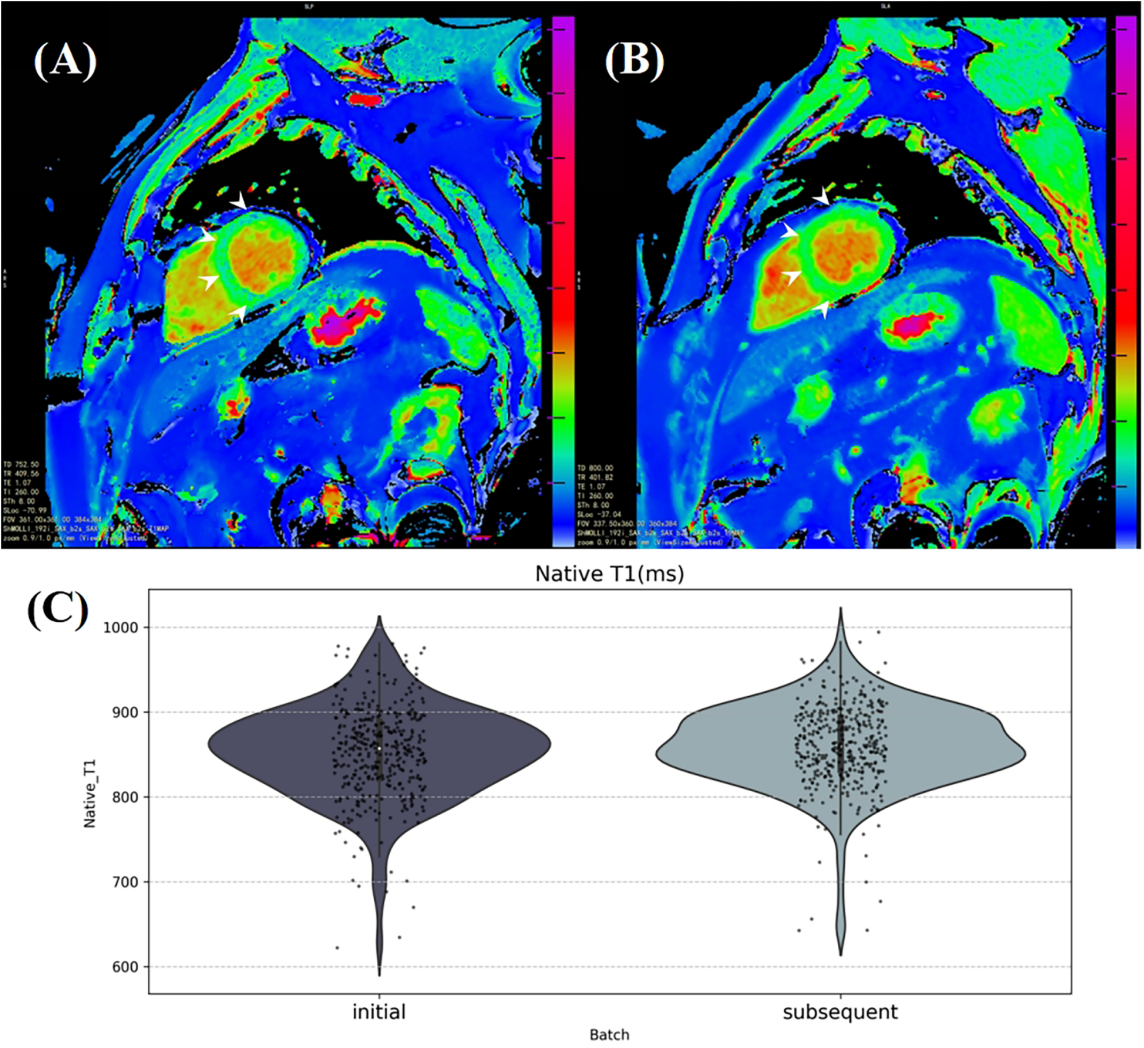
The average myocardial native T1 was calculated from a manually labeling region in a mid-ventricular short axis slice on the T1 mapping. **(A)** and **(B)** illustrate a significant increase in myocardial native T1, particularly in the interventricular septum area (white arrows), in a healthy 53-year-old male participant 185 days after SARS-CoV-2 antibody turned positive. **(C)** The violin plot shows the distribution of average myocardial native T1 in the initial and subsequent CMR batches of SARS-CoV-2 antibody positive participants.

Additionally, in overall seropositive participants (*n* = 392), myocardial native T1 was found to be increased after antibody positivity (mean ± SD: 852.77 ± 53.55 ms vs. 860.01 ± 47.81 ms, *P* = 0.012, Supplementary Table S2, Figure 3).

### 3.3 Variation of cardiac parameters over the interval period

Our investigation aimed to determine whether there were significant changes in cardiac structure and function between SARS-CoV-2 seropositive and seronegative groups over time. We analyzed cardiac parameters from consecutive CMR examinations and calculated the rate of change by dividing the difference between the two CMR measurements by the initial CMR measurement, and expressed as a percentage. We then calculated the interval between the two scans, ultimately deriving the annualized rate of change for the CMR parameters, enabling standardized comparisons.

As shown in Table 4, both SARS-CoV-2 seropositive and seronegative groups exhibited reductions in cardiac function parameters such as LVEF, LVGFi, CO, and CI, as well as increases in cardiac structural parameters such as PWT, RWT, LVM, LVMI, and LVMVR. These results indicate significant changes in these cardiac parameters over an average interval of approximately one hundred days. However, despite similar trends in both groups, the differences between them did not reach statistical significance.

**Table 4:** Annual change rates on CMR parameters of the two groups.

LV parameters	SARS-CoV-2 antibody positive *n* = 261	SARS-CoV-2 antibody negative *n* = 261	*P-*value
LVEDV (%)	−0.795 (16.336)	−1.005 (15.365)	0.801*
LVESV (%)	1.468 (45.363)	0.677 (47.174)	0.796*
LVEDVi (%)	0.007 (18.062)	−1.035 (14.637)	0.472
LVESVi (%)	−0.213 (9.962)	−0.039 (11.252)	0.843
LVSV (%)	0.007 (18.062)	−1.035 (14.637)	0.472
LVEF (%)	−0.213 (9.962)	−0.039 (11.252)	0.843
CO (%)	−3.554 (19.193)	−4.704 (17.560)	0.458
CI (%)	−3.403 (19.237)	−4.494 (17.130)	0.490
LVM (%)	1.067 (12.189)	0.317 (9.552）	0.423
Dd (%)	0.561 (9.421)	−0.719 (7.089)	0.072
Ds (%)	0.984 (13.188)	0.638 (13.619)	0.766
PWT (%)	3.431 (18.345)	1.517 (18.867)	0.228
RWT (%)	4.100 (22.906)	3.059 (21.952)	0.586
LVMi (%)	1.178 (11.768)	0.596 (9.261)	0.512
LVGFi (%)	−0.410 (16.940)	−0.471 (15.848)	0.966
LVMVR (%)	3.219 (20.825)	2.607(17.140)	0.723

Abbreviations as in Table 3.

* Indicates results from the Wilcoxon test.

### 3.4 Association between SARS-CoV-2 antibody positivity and LV hypertrophy incidence

Considering the observed increase in average myocardial native T1 levels following the onset of antibody positivity, indicative of a potential trend towards myocardial fibrosis, we utilized LVMi, a direct indicator derived from CMR imaging, to assess the occurrence of LV hypertrophy.

After excluding 34 participants (17 men and 17 women) who demonstrated LV hypertrophy at their initial CMR examination, logistic regression analysis revealed that SARS-CoV-2 antibody positivity was associated with an increased odds ratio (OR) for the incidence of new LV hypertrophy. The univariate analysis showed an OR of 2.517 [95% CI: (0.838–7.566), *P* = 0.100]. Model 2 adjusting for confounders resulted in an OR of 3.257 [95% CI: (1.036–10.239), *P* = 0.043]. Further adjustment for baseline comorbidities yielded an OR of 2.866 [95% CI: (0.907–9.057), *P* = 0.073]. Detailed results are provided in Table 5.

**Table 5:** Logistic regression analysis of the association between SARS-CoV-2 antibody positivity and the incidence of new LV hypertrophy.

SARS-CoV2 antibody status	OR (95% CI)	*P*-value
Univariate	2.517 (0.838–7.566)	0.100
Adjusted for Confounders	3.257 (1.036–10.239)	0.043
Fully Adjusted	2.866 (0.907–9.057)	0.073

Confounders: age, sex, ethnity, TDI, height, weight, BMI, SBP, DBP.

Fully Adjusted: Confounders + baseline comorbidities including hypertension, cardiovascular diseases and diabetes.

## 4 Discussion

### 4.1 Main findings

In this study, we investigated a cohort of participants who underwent CMR examinations both before and after SARS-CoV-2 antibody testing. Our findings revealed a decrease in CO and CI among participants with positive SARS-CoV-2 antibody status. Although a similar declining trend was observed in the seronegative group, no significant differences were evident in the inter-group comparison of changes in CMR parameters over time. After 1:1 PSM, we found no significant association between SARS-CoV-2 antibody status and the status of LV structure or pumping function. Despite finding subtle intra-group changes, the annual change rates of all LV parameters did not show significant differences between the SARS-CoV-2 seropositive and seronegative groups. This suggests that within the average interval of approximately one hundred days, SARS-CoV-2 antibody positivity might not induce substantial changes in LV structure or function detectable through CMR.

Additionally, we observed an elevation in myocardial native T1 in seropositive participants, indicating a potential impact of SARS-CoV-2 antibody positivity on myocardial fibrosis over a period of approximately 110 days. Logistic regression analysis further revealed that SARS-CoV-2 antibody positivity was associated with an increased risk of LV hypertrophy, with an adjusted odds ratio of 3.257.

### 4.2 Integration into the literature

Our findings align with previous studies reporting cardiovascular sequelae following SARS-CoV-2 infection. For instance, a study involving 148 COVID-19 patients with elevated troponin levels found that 19% had myocardial infarction, with 66% of these cases occurring in individuals without prior coronary disease (16). Similarly, Huang et al. (17) reported elevated LV global T1 and ECV in COVID-19 patients compared to healthy controls, although no significant differences in LV structure or pumping function were observed. These studies, however, were limited to post-infection CMR examinations and primarily included participants with moderate to severe COVID-19, lacking self-comparisons before and after infection.

Follow-up studies at three and six months post-infection have shown gradual improvements in certain cardiopulmonary indicators, such as right ventricular EDV, right ventricular EF, T1, LGE, and peak VO2. However, symptoms like decreased exercise tolerance and muscle fatigue persisted without significant improvement (18). A multi-center prospective cohort study involving 182 COVID-19 patients at three and twelve months follow-ups reported no significant changes in left or right ventricular structure and function, suggesting that cardiac impairments may be reversible in some cases (19). Notably, these studies did not find significant differences in LV parameters during the observation period as well, with potential COVID-19-related effects primarily observed on several right ventricular metrics (20).

In contrast to these studies, our research benefits from the availability of baseline CMR data, allowing us to directly compare cardiac parameters before and after SARS-CoV-2 antibody testing. This unique design minimizes confounding effects and provides a clearer picture of the cardiac impacts associated with SARS-CoV-2 antibody positivity. However, our findings differ from some previous reports, possibly due to the shorter follow-up period and the inclusion of both naturally infected and vaccinated individuals in the seropositive group. This heterogeneity may have diluted the observed differences in cardiac structural and functional changes between seropositive and seronegative individuals.

Given the limitation of the UK Biobank CMR protocol, native T1 values represent one of the most precise indicators available for assessing myocardial changes. A recent study from the UK Biobank CMR cohort found that higher native myocardial T1 was associated with various diseases, such as heart failure, nonischemic cardiomyopathies, atrial fibrillation, stroke, and diabetes (21). Our observation of elevated native T1 in seropositive participants supports the hypothesis that SARS-CoV-2 antibody positivity may contribute to myocardial fibrosis, although further studies using T2 STIR or LGE imaging are needed to exclude the possibility of acute myocarditis-induced myocardial edema as a confounding factor.

The CardioCOVID-Gemelli study investigated the relationship between COVID-19 vaccination status and myocardial injury, finding that vaccination had a protective effect against myocardial injury in elderly individuals (OR: 0.57, 95% CI: 0.34–0.94; *P* = 0.03) but was an independent risk factor in younger individuals (OR: 4.44, 95% CI: 1.28–15.34, *P* = 0.02) (22). While our study did not specifically differentiate between natural infection and vaccination, the observed increase in LVMi suggests that SARS-CoV-2 antibody positivity may accelerate cardiac remodeling.

Unlike most observational COVID-19 studies, which lack baseline cardiac data, our study is the first to utilize two sets of CMR images, including examinations before and after SARS-CoV-2 antibody test, from the UK Biobank cohort. Specifically, we selected participants who underwent their initial CMR examinations after 2019 but before their SARS-CoV-2 exposure to minimize the confounding effects of other factors on cardiac data. This approach allows us to explore the association between SARS-CoV-2 antibody status and variations in LV structure and function over an average interval of 110 days. By comparing direct LV parameters across different serological immune states, we provide a unique perspective on the cardiac impacts of SARS-CoV-2 antibody positivity.

Furthermore, our findings showed reductions in cardiac parameters such as LVEF, CO, and CI, as well as increases in cardiac structural parameters like LVMi, LVESVi, Ds, PWT, and LVMVR, in both SARS-CoV-2 antibody-positive and antibody-negative groups. These changes may reflect the natural cardiac remodeling process that occurs over time, particularly given the approximately 100-day interval between the initial and follow-up CMR scans. This suggests that both groups experienced alterations in cardiac function and structure during this period, regardless of antibody status. The observed reductions in cardiac function and increases in structural measures could indicate early signs of cardiac remodeling that may be independent of SARS-CoV-2 exposure.

### 4.3 Limitation

Several limitations should be acknowledged. First, SARS-CoV-2 antibody positivity was determined using serological antibody tests, but positive results could not differentiate between natural infection and successful vaccination, which may have different effects on cardiac structure and function. Similarly, negative antibody results could not distinguish between uninfected individuals and those with a non-responsive vaccination. Second, our study required a second CMR scan to assess cardiac changes after a clear positive or negative antibody test result, leading to potential participant loss for those who only had baseline images available. The average follow-up period of approximately 100 days may not be long enough to fully capture the long-term cardiovascular effects of COVID-19.

Additionally, due to safety and practical considerations, the UK Biobank CMR protocol did not include enhanced CMR imaging, such as T2 STIR sequences, LGE, and ECV analyses. As a result, we could not exclude the possibility that myocardial edema contributed to the observed Native T1 changes. Finally, since all raw images were obtained from the UK Biobank, the CMR follow-up examinations were part of a general survey, and specific clinical indications for these examinations were not available.

Furthermore, while an ICC analysis showed good consistency between the three observers, the varying experience levels of the observers could have introduced some bias. This potential variability is acknowledged as a limitation.

## 5 Conclusion

Our findings suggest subtle cardiac changes associated with SARS-CoV-2 antibody positivity over an approximately 100-day period, including decreases in CO and CI, an increase in myocardial native T1, and a potential risk of LV hypertrophy. However, no significant differences were observed in LV structure and function between the seropositive and seronegative groups. These results are exploratory, and further studies with longer follow-up are needed to clarify the long-term cardiovascular effects of SARS-CoV-2 antibody positivity.

## Data Availability

The datasets presented in this article are not readily available because the raw imaging data and non-imaging participant characteristics are available from the UK Biobank. Requests to access the datasets should be directed to http://www.ukbiobank.ac.uk/register-apply.
