# Supplementary material for: Impact of self-reported SARS-CoV-2 antibody positivity on cardiac structure and function: findings from UK Biobank CMR cohort
Source: Front Cardiovasc Med. 2025 Feb 27;12:1462263. doi: 10.3389/fcvm.2025.1462263 (PMC11903740; doi:10.3389/fcvm.2025.1462263)
Supplement: Supplementary file 1 [file Datasheet1.docx]

**Supplementary Table 1. International Classification of Disease (ICD) codes used to define various disease of baseline characteristic.**

| **Disease** | **ICD code** | **Code description** |
| --- | --- | --- |
| Hypertension | I10 | Essential (primary) hypertension |
| Hypertension | I15 | Secondary hypertension |
| High Cholesterol | E78.0 | Pure hypercholesterolaemia |
| High Cholesterol | E78.1 | Pure hyperglyceridaemia |
| High Cholesterol | E78.2 | Mixed hyperlipidaemia |
| High Cholesterol | E78.3 | Hyperchylomicronaemia |
| High Cholesterol | E78.4 | Other hyperlipidaemia |
| High Cholesterol | E78.5 | Hyperlipidaemia, unspecified |
| Diabetes Mellitus | E10 | Insulin-dependent diabetes mellitus |
| Diabetes Mellitus | E11 | Non-insulin-dependent diabetes mellitus |
| Diabetes Mellitus | E12 | Malnutrition-related diabetes mellitus |
| Diabetes Mellitus | E13 | Other specified diabetes mellitus |
| Diabetes Mellitus | E14 | Unspecified diabetes mellitus |
| Asthma | J45 | Asthma |
| Asthma | J46 | Status asthmaticus |
| Cardiac diseases | I20 | Angina pectoris |
| Cardiac diseases | I21 | Acute myocardial infarction |
| Cardiac diseases | I22 | Subsequent myocardial infarction |
| Cardiac diseases | I23 | Certain current complications following acute myocardial infarction |
| Cardiac diseases | I24 | Other acute ischaemic heart diseases |
| Cardiac diseases | I25 | Chronic ischaemic heart disease |
| Cardiac diseases | I30 | Acute pericarditis |
| Cardiac diseases | I31 | Other diseases of pericardium |
| Cardiac diseases | I32 | Pericarditis in diseases classified elsewhere |
| Cardiac diseases | I33 | Acute and subacute endocarditis |
| Cardiac diseases | I34 | Nonrheumatic mitral valve disorders |
| Cardiac diseases | I35 | Nonrheumatic aortic valve disorders |
| Cardiac diseases | I36 | Nonrheumatic tricuspid valve disorders |
| Cardiac diseases | I37 | Pulmonary valve disorders |
| Cardiac diseases | I38 | Endocarditis, valve unspecified |
| Cardiac diseases | I39 | Endocarditis and heart valve disorders in diseases classified elsewhere |
| Cardiac diseases | I40 | Acute myocarditis |
| Cardiac diseases | I41 | Myocarditis in diseases classified elsewhere |
| Cardiac diseases | I42 | Cardiomyopathy |
| Cardiac diseases | I43 | Cardiomyopathy in diseases classified elsewhere |
| Cardiac diseases | I44 | Atrioventricular and left bundle-branch block |
| Cardiac diseases | I45 | Other conduction disorders |
| Cardiac diseases | I46 | Cardiac arrest |
| Cardiac diseases | I47 | Paroxysmal tachycardia |
| Cardiac diseases | I48 | Atrial fibrillation and flutter |
| Cardiac diseases | I49 | Other cardiac arrhythmias |
| Cardiac diseases | I50 | Heart failure |

**Supplementary Table 2. Comparison of average myocardial native T1 values in SARS-CoV-2 seropositive patients before and after SARS-CoV-2 exposure.**

|  | Initial CMR n=392 | Subsequent CMR n=392 | *P* value |
| --- | --- | --- | --- |
| Native T1 (ms) | 852.77(53.55) | 860.01(47.81) | 0.012 |

Independent variables was calculated as means and standard deviation.
